# Are exercise programs relevant in psychiatric wards?

**DOI:** 10.1192/j.eurpsy.2022.1613

**Published:** 2022-09-01

**Authors:** I. Valada, I. Caldas, S. Vieira, I. Pereira

**Affiliations:** 1 Centro Hospitalar Psiquiátrico de Lisboa, Clínica 4, Lisboa, Portugal; 2 Centro Hospitalar Psiquiátrico de Lisboa, Clínica 2, Lisboa, Portugal; 3 Centro Hospitalar Psiquiátrico de Lisboa, Clínica 6, Lisboa, Portugal

**Keywords:** physical activity, exercise program, exercise, mental health care

## Abstract

**Introduction:**

About 3% of the general population suffers from Severe Mental Illness (SMI), including schizophrenia spectrum, bipolar and major depressive disorders. In this group, the rates of cardiovascular disease, diabetes mellitus and metabolic syndrome are approximately twice as high as the general population and the life expectancy is 13-30 years inferior compared to age and sex matched controls, greatly due to medical comorbidities. Low levels of physical activity (PA) and low fitness are likely to play a role.

**Objectives:**

To review the evidence about the effects of PA on physical health markers and psychiatric clinical symptoms of SMI patients.

**Methods:**

We performed a literature review on the impact of exercise programs in physical and mental health of SMI patients using the PubMed and Google Scholar databases.

**Results:**

Several studies demonstrate that PA improves a variety of physical health markers in SMI patients, such as body weight, body mass index, waist circumference, body fat percentage, cardiorespiratory fitness, systolic blood pressure and HDL cholesterol. In parallel, there is evidence to support the benefit of PA in mental health, especially due to its effect on mood. Furthermore, PA promotes cognitive functioning, sleep quality, quality of life, self-esteem and fosters social interaction. Moreover, in patients with schizophrenia, exercise seems to decrease negative symptoms.

**Conclusions:**

Implementation of regular physical activities in psychiatric wards should be considered whenever possible, due to its positive effects on physical and mental health. The adoption of structured exercise programs in psychiatric wards is feasible, safe, and well-received by patients.

**Disclosure:**

No significant relationships.

